# Hormone receptor expression patterns define clinically meaningful subgroups of endometrioid ovarian carcinoma

**DOI:** 10.1016/j.ygyno.2019.09.001

**Published:** 2019-11

**Authors:** Robert L. Hollis, Barbara Stanley, Yasushi Iida, John Thomson, Michael Churchman, Tzyvia Rye, Melanie Mackean, Fiona Nussey, Charlie Gourley, C. Simon Herrington

**Affiliations:** a Nicola Murray Centre for Ovarian Cancer Research, Cancer Research UK Edinburgh Centre, MRC Institute of Genetics and Molecular Medicine, University of Edinburgh, UK; b Department of Obstetrics and Gynecology, The Jikei University School of Medicine, Minato-ku, Tokyo, Japan; c Edinburgh Cancer Centre, Western General Hospital, Edinburgh, UK

**Keywords:** Endometrioid ovarian carcinoma, Progesterone receptor, Estrogen receptor, Androgen receptor, Ovarian cancer, Body mass index

## Abstract

**Background:**

Numerous studies have investigated the association between hormone receptor expression and clinical outcome in ovarian carcinoma (OC); however, these have largely focussed on serous OCs, with few studies reporting specifically on endometrioid OCs (EnOC). Where analyses have been stratified by histotype, expression has been assessed using the percentage of positive tumor cells, without accounting for nuclear expression intensity.

**Methods:**

Here we assess the expression levels of progesterone receptor (PR), estrogen receptor alpha (ER) and androgen receptor (AR) using histoscore – a nuclear scoring method incorporating both proportion of positive cells and the intensity of nuclear staining – across a cohort of 107 WT1 negative EnOCs.

**Results:**

Hierarchical clustering by PR, ER and AR histoscores identified four EnOC subgroups (PR+/ER+, PR+/ER−, PR−/ER+ and PR−/ER−). EnOC patients in the PR+/ER+ and PR+/ER− groups displayed favorable outcome (multivariable HR for disease-specific survival 0.05 [0.01–0.35] and 0.05 [0.00–0.51]) compared to the PR−/ER+ group. Ten-year survival for stage II PR^high^ and PR^low^ cases was 94.1% and 42.4%. ER^high^ EnOC patients (PR+/ER+, PR−/ER+) had higher body mass index compared to ER^low^ cases (*P* = 0.015) and high grade serous OC patients (*P* < 0.001).

**Conclusion:**

These data demonstrate that endometrioid OC cases with high PR expression display markedly favorable outcome. Stage II EnOCs with high PR expression represent potential candidates for de-escalation of first-line therapy. Future work should seek to characterise the sensitivity of PR and ER positive EnOCs to endocrine therapy.

## Highlights

• We identify four subgroups of endometrioid ovarian carcinoma (EnOC) defined by hormone receptor expression patterns.
• EnOC patients in the PR^high^ (PR+/ER+, PR+/ER−) groups demonstrate markedly favorable outcome.
• Stage II EnOC patients in the PR^high^ groups display a ten-year survival of approximately 95%.
• ER^high^ (PR+/ER+, PR−/ER+) EnOC patients had a higher body mass index vs ER^low^ cases and high grade serous carcinoma patients.

## 1 Introduction

Ovarian cancer is the most lethal cancer of the female genital tract, and accounts for over 180,000 deaths per year worldwide [1]. The vast majority of cases are ovarian carcinomas (OCs), comprising five core histological subtypes: high grade serous (HGS), endometrioid, clear cell, low grade serous (LGS) and mucinous OC [2].

Endometrioid OCs (EnOCs) account for approximately 10% of cases, are associated with favorable prognosis compared to other histotypes, and are often diagnosed at FIGO stage I or II [[2], [3], [4]]. EnOCs are frequently estrogen receptor alpha (ER) and/or progesterone receptor (PR)-positive, and display WT1 negativity [5]. The routine use of WT1 immunohistochemistry (IHC) is known to improve the reproducibility of EnOC diagnosis, aiding the distinction of these cases from HGS or LGS OCs which can demonstrate morphological resemblance to EnOC [6,7].

Reports of the prognostic impact of hormone receptor expression in OC have largely focussed on serous cases, with studies almost ubiquitously focusing on either these tumors alone [[8], [9], [10], [11]], or mixed patient populations dominated by OCs of serous histology [[12], [13], [14], [15], [16], [17]]. Mixed histology studies interrogating survival without stratifying by histological subtype have the potential to be confounded by the known differential expression of hormone receptors across these subtypes [6,18], which are now recognised to demonstrate markedly distinct clinical outcome [3,4].

A number of studies have included EnOC cases when investigating the prognostic impact of hormone receptor positivity on OC outcome [[13], [14], [15], [16],[19], [20], [21], [22]]; however, the majority of these studies have included fewer than 30 EnOC cases in the context of mixed histology cohorts [[14], [15], [16],^19^,^20^]. One study collected a relatively large series of EnOCs (84 cases), but did not perform histotype-specific analysis of outcome [13]. The Ovarian Tumor Tissue Analysis Consortium investigated the prognostic impact of ER and PR expression specifically in EnOC, where investigators used a three-tier taxonomy for quantifying expression magnitude, reporting a survival benefit in ER-positive and PR-positive cases [21], which was mirrored in an independent study of from Rambau et al. [22]. However, as with many other investigations of hormone receptor expression in OC, these studies defined positivity by the proportion of marker-positive tumor cells (as <1%, 1–50% and ≥50% positive tumor nuclei) without accounting for intensity of nuclear staining. Quantification of both the proportion of positive cells and the intensity of nuclear expression may allow for a more granular assessment of expression patterns. Notably, the most widely used scoring systems for hormone receptor positivity incorporate both of these measures [23].

Androgen receptor (AR) expression has also been associated with favorable outcome in some OC cohorts [11,14,17]; however, the clinical impact of AR expression specifically in EnOC is poorly defined.

Histoscore is a nuclear staining intensity scoring method which incorporates both the proportion of positive tumor nuclei and the intensity of nuclear staining [24]. We and others have demonstrated the utility of ER histoscore for identifying OCs that derive the greatest benefit from endocrine therapy, and this method is routinely used to identify good candidates for endocrine treatment regimens locally within our centre [[25], [26], [27]].

Here we report on hormone receptor expression across a cohort of EnOCs identified following contemporary pathology review utilizing immunohistochemistry (IHC) for WT1. We quantify expression of ER, PR and AR using histoscore in order to determine the prognostic impact of hormone receptor expression patterns specifically in EnOC.

## 2 Materials and methods

### 2.1 Cohort collection

We recently identified a cohort of 107 WT1 negative EnOCs following contemporary pathology review of OC cases (*n* = 289) with documented endometrioid histology from the Edinburgh Ovarian Cancer Database (full data to be reported elsewhere). Tumor material was reviewed using H&E stained slides and IHC with antibodies for WT1 (DAKO, clone 6F-H2; 1:1000 dilution) and p53 (DAKO, clone DO-7; 1:50 dilution); metastases from primary uterine carcinomas, as defined by clinicopathological criteria [28], were excluded. Use of human tumor tissue for research was approved by South East Scotland Human Annotated BioResource (Lothian NRS Bioresource ethics reference 15/ES/0094-SR494). Demographics of the 107 WT1 negative EnOCs are summarised in Table 1.

**Table 1:** Demographics of 107 WT1 negative EnOCs.

		N	%/range
Age at diagnosis	Median years	57	28–88
Body mass index	Median	25.5	18.0–44.0
Disease grade	G1 EnOC	80	74.7
	G2 EnOC	19	17.8
	G3 EnOC	8	7.5
FIGO stage at diagnosis	I	47	44.8
	II	42	40.0
	III	10	9.5
	IV	6	5.7
	NA	2	
RD following debulking	<2 cm^a^	93	90.3
	≥2 cm	10	9.7
	NA	4	
p53 IHC pattern	AP	11	10.5
	AN	0	0.0
	WT	94	89.5
	NE	2	

EnOC, endometrioid ovarian carcinoma; NA, not available; RD, residual disease; IHC, immunohistochemistry; AP, aberrant diffuse nuclear positive; AN, aberrant null; WT, wild-type; NE, not evaluable.

a Due to the retrospective nature of this cohort and the historic classification of <2 cm residual disease as optimal debulking, debulking status is not resolved beyond <2 cm in this cohort.

### 2.2 Clinical annotation

Correlation of molecular data to clinicopathological variables and clinical outcome in patients with ovarian carcinoma was approved by NHS Lothian Research and Development (reference 2007/W/ON/29). Clinical data were retrieved from the Edinburgh Ovarian Cancer Database, wherein data regarding the diagnosis and management of ovarian cancer patients treated at the Edinburgh Cancer Centre are entered prospectively as part of routine care, alongside archived patient records. Body mass index (BMI) was calculated using patient height and weight as recorded at time of first chemotherapy administration. Adjuvant treatment regimens and staging information is summarised in Table S1. Patients underwent 3-monthly clinical follow-up for 2 years and 6-monthly follow-up for a further 3 years, after which patients were tracked via yearly contact with their general practitioner to determine patient status. Radiological imaging was used to determine relapse upon a significant rise in CA125 or if patients presented with symptomatic deterioration.

### 2.3 Immunohistochemical staining for PR, ER and AR

A human tissue microarray (TMA) was constructed to assess tumor cell positivity for markers of interest. TMAs were constructed using H&E stained slides, marked to identify tumor area by an expert pathologist (CSH), to guide tissue coring. Three 0.8 mm tissue cores were taken per EnOC case.

IHC for PR was performed using 1:50 mouse anti-human PR antibody M3569 (DAKO, clone PgR-636). ER staining was performed using 1:50 dilution of rabbit anti-human ER antibody M3643 (DAKO, clone EP1). AR staining was performed using 1:50 dilution of mouse anti-human AR antibody M3562 (DAKO, clone AR441). IHC was performed using the Leica BOND III Autostainer with epitope retrieval solution 2 for 20 min. Normal human breast tissue was used as a positive control for ER and PR, while normal human prostate tissue was used as a positive control for AR. Negative controls were performed using further sections without the addition of primary antibody.

### 2.4 Histoscoring of PR, ER and AR immunohistochemical staining

Marker positivity was evaluated by histoscore, a nuclear staining scoring method incorporating both proportion of positive cells and the intensity of nuclear positivity [24]. The proportion of tumor cells in each samples scored as 0 (negative), 1+ (weak positive), 2+ (moderately positive), or 3+ (strong positive) was recorded, using validated breast carcinoma control samples for comparison. Per-core histoscore was calculated as 1×(%cells 1+) + 2×(%cells 2+) + 3×(%cells 3+) (Fig. S1).

Each core was scored by two independent observers. Comparison of histoscore assessment between the two observers demonstrated excellent agreement (weighted Kappa >0.90 for all markers; Spearman's ρ ≥ 0.85, *P* < 0.001) (Table S2).

Per-patient histoscore was calculated as the mean of evaluable cores, weighted by percentage tumor present in each core (see Appendix A). Cores with <10% tumor cellularity were excluded from analysis. Patients with fewer than two assessable cores were considered non-evaluable for marker expression.

### 2.5 Statistical analysis

Statistical analyses were performed using R version 3.5.1. Relapse-free survival (RFS) was calculated as time from diagnosis to first radiological relapse. Comparisons of frequency were made using the Fisher's exact test and Chi-squared test, as appropriate. Data modality and normality was assessed by Hartigan's Dip test and the Shapiro-Wilk test. Comparisons of continuous variables were made using the unpaired *t*-test and Mann Whitney-*U* test, as appropriate.

Survival differences were assessed in the survival R package [29] using Cox proportional hazards regression models and visualised using the Kaplan-Meier method. Subgroups of EnOC were identified by hierarchical clustering of PR, ER and AR histoscores using Euclidian distance and Ward's Linkage. Correction for multiplicity of testing was performed using the Bonferroni method, where specified.

## 3 Results

### 3.1 Patterns of ER, PR and AR expression in EnOC

56.3% and 48.3% of cases demonstrated PR histoscore of ≥100 and ≥200. For ER, 60.9% and 29.9% of cases displayed a histoscore of ≥100 and ≥200. 13.2% and 3.3% of cases demonstrated an AR histoscore of ≥100 and ≥200. Hormone receptor histoscores across the EnOC cohort are summarised in Fig. S2 and Table S3. Expression distribution was non-normal for all markers (Shapiro-Wilk *P* < 0.001 for PR, ER and AR), and PR expression demonstrated a bimodal distribution (Fig. S2A) (Hartigan's Dip test, *P* < 0.0001).

Expression of PR and ER demonstrated highly significant correlation (Spearman's ρ = 0.60, P < 0.0001). Expression of PR and AR demonstrated weaker correlation that was also statistically significant (ρ = 0.34, *P* = 0.002), while ER and AR demonstrated significant correlation of intermediate magnitude (ρ = 0.45, *P* < 0.001).

### 3.2 PR/ER/AR-defined subgroups of EnOC

**Fig. 1:**
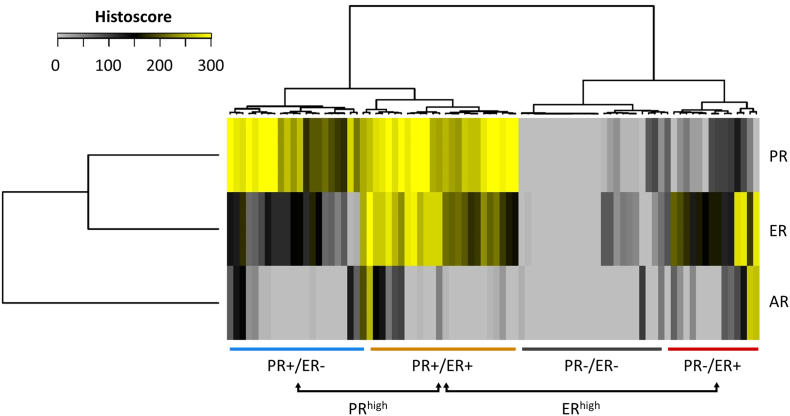
Hierarchical clustering by PR, ER and AR histoscores identifies subgroups of EnOC.

84 cases had evaluable histoscores for all three markers (*n* = 15 not available [NA] for PR/ER/AR; *n* = 2 NA for ER/PR; *n* = 3 NA for PR; n = 3 NA for ER). Hierarchical clustering of EnOC cases using their PR, ER and AR histoscores identified subgroups of EnOC based on hormone receptor expression patterns (Fig. 1). Four major subgroups were identified, defined largely by patterns of PR and ER expression: PR+/ER− (*n* = 21), PR+/ER+ (*n* = 25), PR−/ER+ (*n* = 14) and PR−/ER− (*n* = 24).

AR expression appeared to contribute little toward subgroup classification, likely owing to the almost ubiquitous low expression of AR across the cohort. However, AR histoscore was significantly lower in the PR−/ER− group versus the other EnOC groups (Bonferroni-adjusted *P* < 0.01 for all comparisons) (Fig. S3, Table S4).

**Fig. 2:**
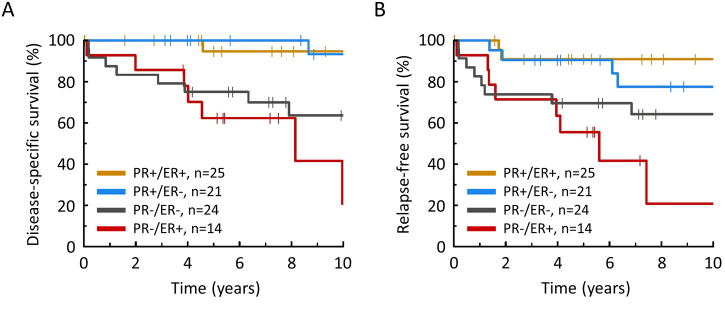
Disease-specific (A) and relapse-free (B) survival of EnOC subgroups.

The PR^high^ groups (PR+/ER+ and PR+/ER−) demonstrated favorable DSS (HR = 0.11, 95% CI 0.02–0.54 and 0.05, 95% CI 0.00–0.44, respectively) compared to the PR−/ER+ group, with the PR−/ER− group showing intermediate prognosis (HR = 0.50, 95% CI 0.18–1.40) (Fig. 2A and Table S5). These data were mirrored upon investigation of RFS (Fig. 2B, Table S5). Within stage II cases specifically, 10-year DSS in the PR^high^ (*n* = 32) and PR^low^ cases (*n* = 15) was 94.1% and 42.4%, respectively (HR = 0.10, 95% CI 0.01–0.84) (Fig. S4).

### 3.3 Association of EnOC subgroups with clinicopathological features in EnOC

Clinicopathological features of the four EnOC subgroups is summarised in Table 2. The PR^high^ groups (PR+/ER+ and PR+/ER−) appeared to comprise a greater portion EnOCs with grade I histology (*P* = 0.023), early stage (FIGO I/II) at diagnosis (95.5% vs 81.6%, *P* = 0.074) and wild-type p53 expression pattern (*P* = 0.042). However, these were not significant following correction for multiple testing (Bonferroni-adjusted *P* = 0.070, *P* = 0.222 and *P* = 0.127). The PR+/ER− group appeared to demonstrate younger age at diagnosis, but this did not reach statistical significance (*P* = 0.055).

**Table 2:** Demographics of EnOC subgroups.

		Group				*P*-value
		PR+/ER+	PR+/ER−	PR−/ER+	PR−/ER−	
Age at diagnosis	Median	59.0	54.0	60.5	60.5	0.055^a^
BMI	Median	27.2	23.4	28.9	24.1	0.015^b^
Grade	G1 EnOC	21 (84.0%)	18 (85.7%)	7 (50.0%)	16 (66.7%)	0.023^c^
	G2 EnOc	2 (8.0%)	2 (9.5%)	5 (35.7%)	5 (20.8%)	
	G3 EnOc	2 (8.0%)	1 (4.8%)	2 (14.3%)	3 (12.5%)	
FIGO stage at diagnosis	I	11 (45.8%)	10 (50.0%)	6 (42.8%)	10 (41.7%)	0.074^d^
	II	11 (45.8%)	10 (50.0%)	6 (42.8%)	9 (37.5%)	
	III	2 (8.3%)	0 (0.0%)	1 (7.1%)	3 (12.5%)	
	IV	0 (0.0%)	0 (0.0%)	1 (7.1%)	2 (8.3%)	
	NA	1	1	0	0	
RD following debulking	<2 cm	22 (91.7%)	20 (100.0%)	12 (92.3%)	21 (91.3%)	0.653^e^
	≥2 cm	2 (8.3%)	0 (0.0%)	1 (7.7%)	2 (8.7%)	
p53 IHC pattern	AP	0 (0.0%)	1 (4.8%)	3 (21.4%)	3 (13.0%)	0.042^f^
	AN	0 (0.0%)	0 (0.0%)	0 (0.0%)	0 (0.0%)	
	WT	24 (100.0%)	20 (95.2%)	11 (78.6%)	20 (87.0%)	
	NE	1	0	0	1	

BMI, body mass index; EnOC, endometrioid ovarian carcinoma; NA, not available; RD, residual disease; IHC, immunohistochemistry; AP, aberrant diffuse nuclear positive; AN, aberrant null; WT, wild-type; NE, not evaluable.

a T test; PR+/ER− versus others.

b Mann Whitney-U test: ER^high^ vs ER^low^.

c χ^2^ test, PR^high^ (PR+/ER+ and PR+/ER−) vs PR^low^ (PR−/ER+ and PR−/ER−): low grade (G1) versus high grade (G2/3).

d Fisher's exact test, PR^high^ vs PR^low^: early (FIGO I/II) vs advanced stage (FIGO III/IV).

e Fisher's exact test, PR^high^ vs PR^low^.

f Fisher's exact test, PR^high^ vs PR^low^: P53 aberrant vs wild-type pattern.

Due to the apparent differential distribution of clinicopathological features between the EnOC subgroups, a multivariable analysis for DSS was performed to account for age, stage, grade and residual disease following surgical debulking. In this model, the PR^high^ groups (PR+/ER+ and PR+/ER−) demonstrated significantly favorable DSS (HR = 0.05, 95% CI 0.01–0.35 and 0.05, 95% CI 0.00–0.51, respectively) compared to the PR−/ER+ group, with the PR−/ER− group showing intermediate prognosis (HR = 0.24, 95% CI 0.06–0.99) (Tables S6 and S7).

### 3.4 Correlation of ER status in EnOC with body mass index at patient presentation

**Fig. 3:**
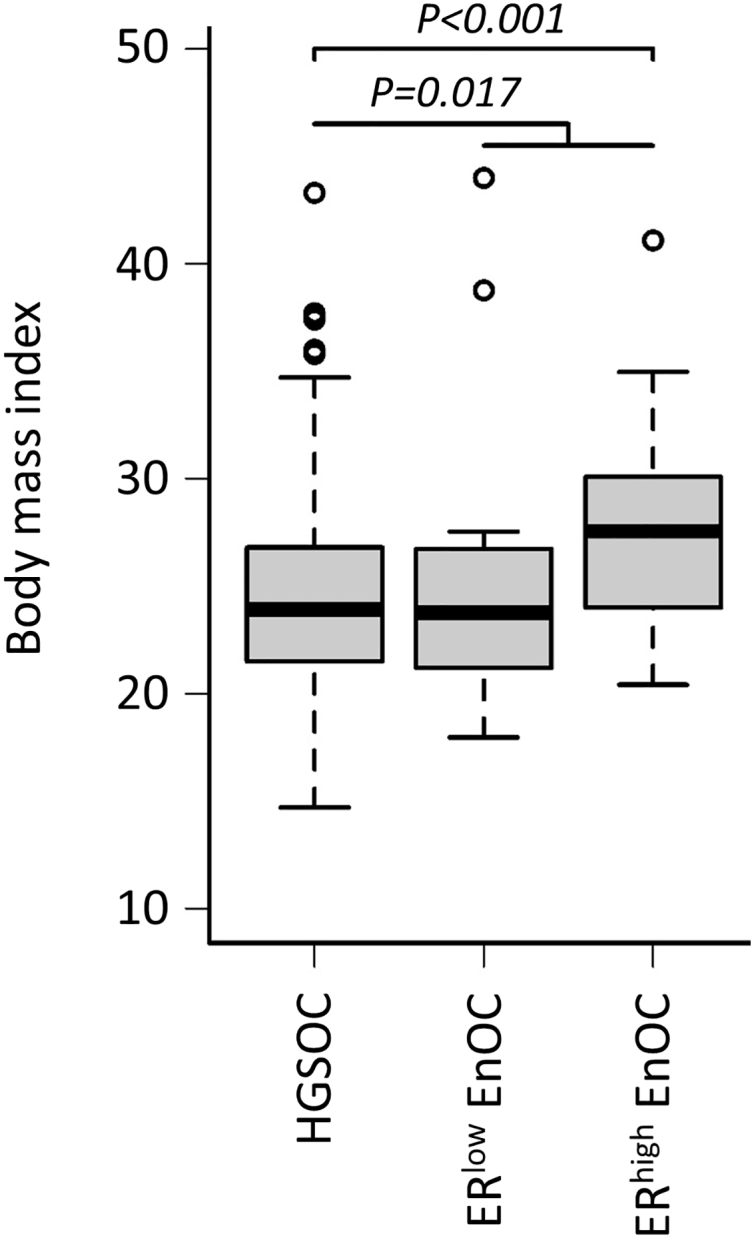
Body mass index of EnOC versus high grade serous OC patients.

We have previously reported a series of 265 HGS OC cases from Edinburgh following contemporary pathology review [30]. The EnOC cohort had higher BMI at first-line chemotherapy initiation compared to the HGS OC comparator cohort (median 25.5 vs 23.9, *P* = 0.017) (Fig. 3). Within the EnOCs, ER^high^ (PR+/ER+, PR−/ER+) cases had a higher BMI compared to ER^low^ cases (median 27.6 vs 23.8, *P* = 0.015), whose BMI was akin to that of the HGS OC cohort (median BMI 23.9, *P* = 0.8768). Accordingly, ER^high^ EnOCs demonstrated markedly higher BMI versus the HGS OC cases (*P* < 0.001; Bonferroni-adjusted *P* = 0.002).

## 4 Discussion

There has been significant research interest in the clinical impact of hormone receptor expression in OC, with regard to both patient survival [[8], [9], [10], [11], [12], [13], [14], [15], [16], [17],^22^] and sensitivity to endocrine therapy [[25], [26], [27]]. Findings relating PR, ER and AR expression to clinical outcome have been mixed, with studies typically performing analysis on heterogeneous OC cohorts without stratifying by histological subtype of disease, which are now known to represent distinct clinical and molecular disease entities [3]. Moreover, methodologies for determining positivity have also been heterogeneous, with many studies classifying specimens only by the proportion of positive tumor cells, without accounting for the intensity of positive nuclear staining.

Here, we describe a cohort of EnOCs identified through contemporary review of all available OCs diagnosed with endometrioid histology at the Edinburgh Cancer Centre, utilizing IHC for WT1 to improve the fidelity of EnOC diagnosis. We assess positivity for PR, ER and AR across 107 EnOCs – all of which were WT1 negative – using histoscore, a scoring method incorporating both the percentage of positive nuclei and the intensity of nuclear staining. Critically, we avoid sample classification by arbitrarily defined positivity thresholds, instead performing clustering of EnOCs based on their PR, ER and AR histoscores in order to identify subgroups of disease defined by patterns of hormone receptor expression. EnOC specimens displayed a low frequency of AR positivity, and AR histoscore appeared to contribute little toward the assignment of samples to the four identified subgroups.

EnOC cases demonstrating high levels of PR (PR+/ER+ and PR+/ER− groups) displayed markedly favorable clinical outcome. These data are consistent with the prolonged survival described in EnOCs with >1% PR-positive nuclei by the Ovarian Tumor Tissue Analysis consortium [21], identifying 10-year patient survival of around 80%, 60–65% and 50% in those demonstrating ≥50%, 1–50% and <1% PR positive tumor cell nuclei. The scoring method utilized in our study – incorporating both percentage positive cells and the nuclear staining intensity using histoscore – may have contributed toward the identification of a PR^high^ EnOC population with exceptional 10-year survival of >90% in this EnOC cohort, providing greater granularity for distinction of tumors demonstrating moderate or strong marker positivity. While PR positivity appeared anti-correlated with features associated with poor prognosis (advanced stage at diagnosis, disease grade and patient age), multivariable analysis identified PR as an independent factor associated with prognosis.

Perhaps most notably, PR^high^ stage II cases demonstrated a 10-year survival of approximately 95% compared to around 40% in the corresponding PR^low^ cases. These data suggest that stage II EnOC with high PR expression may be good candidates for de-escalation of adjuvant chemotherapy regimens to endocrine therapy or observation following primary surgical debulking.

For both PR and ER, the threshold for cluster separation during subgroup identification appeared to lie at a histoscore of approximately 150, and this histoscore threshold could readily be implemented to routinely stratify EnOC management and improve patient prognostication. We recently reported that HGS OCs with strong ER positivity represent those that derive the greatest benefit from endocrine therapy [26], and similar investigations are now warranted to better define the utility of endocrine therapies specifically in EnOC. Defining the efficacy of endocrine therapy in PR^high^ EnOC may further highlight potential opportunities to de-escalate first-line therapy for cases diagnosed with stage II disease.

A number of studies have suggested that ER-positive OCs demonstrate favorable outcome compared to ER-negative cases [9,11,13,15,16], including a large histotype-specific study of EnOC [21]. We demonstrate highly significant correlation between PR and ER histoscore in our EnOC cohort, but did not identify a survival benefit in the PR−/ER+ group. Together, these data suggest that high ER expression may not be independently associated with better outcome in EnOC and that its association with PR expression may explain the apparent survival advantage for ER-positive EnOC patients upon univariable analysis. Indeed, in our cohort, the PR−/ER+ group demonstrated the least favorable outcome.

Upon investigation of BMI, the EnOC cohort appeared to demonstrate higher BMI versus the HGS OC comparator cohort from Edinburgh. However, upon closer investigation, ER^high^ EnOCs demonstrated significantly higher BMI, while ER^low^ EnOCs were comparable to HGS OCs. These data suggest that increased BMI may be associated with development of ER^high^ EnOC. Notably, higher BMI is known to be associated with increased risk of endometrial cancer, the majority of which display endometrioid histology and are considered hormone-driven [31].

## 5 Conclusions

Together, these data add to the growing evidence that hormone receptors represent clinically meaningful biomarkers of patient outcome in OC. Optimal first-line management strategies for patients with PR^high^ EnOC – who appear to experience exceptional long-term survival – may now warrant re-evaluation, particularly in the context of patients diagnosed with stage II disease who would typically undergo systemic chemotherapy [32]. The relative efficacy of endocrine therapy in subgroups of EnOCs and other OC histotypes should also be investigated following the recent demonstration of greatest benefit in HGS OCs demonstrating high ER expression [26].

Future studies should seek to define how expression levels of hormone receptors – particularly PR and ER – overlap with other molecularly-defined subgroups of OC. Within HGS OCs, comparison versus *BRCA1/2* mutation status may be of particular interest, given the association of these events with improved clinical outcome and sensitivity to DNA damaging agents and poly-(ADP-ribose) polymerase (PARP) inhibitors [[33], [34], [35]]. Indeed, there has already been research interest in assessing such co-occurrence [21]. Critically, these analyses will need to be performed in an OC histotype-specific manner, owing to the distinct molecular landscape demonstrate by each of these tumor types.

## Appendix A Supplementary data

Supplementary material Image 1
