# Supplementary material for: Hormone receptor expression patterns define clinically meaningful subgroups of endometrioid ovarian carcinoma
Source: Gynecol Oncol. 2019 Nov;155(2):318–23. doi: 10.1016/j.ygyno.2019.09.001 (PMC6854467; doi:10.1016/j.ygyno.2019.09.001)
Supplement: Supplementary file 1 — Supplementary material [file mmc1.docx]

Supplementary materials

Appendix A

For cores where observer 1 and observer 2 disagreed on histoscoring (histoscore difference ≥50), a third observer evaluated histoscore independently, and a consensus histoscore for the core was then reached between the three observers.

Per-patient histoscore was calculated using the following formula, weighting the histoscore calculation toward cores with greater proportion of tumour cells:

A = %age tumour of core 1; B = %age tumour of core 2; C = %age tumour of core 3

Per-patient histoscore =

$$\frac{(core 1 Histoscore x A) + (core 2 Histoscore x B) + (core 3 Histoscore x C)}{A+B+C}$$

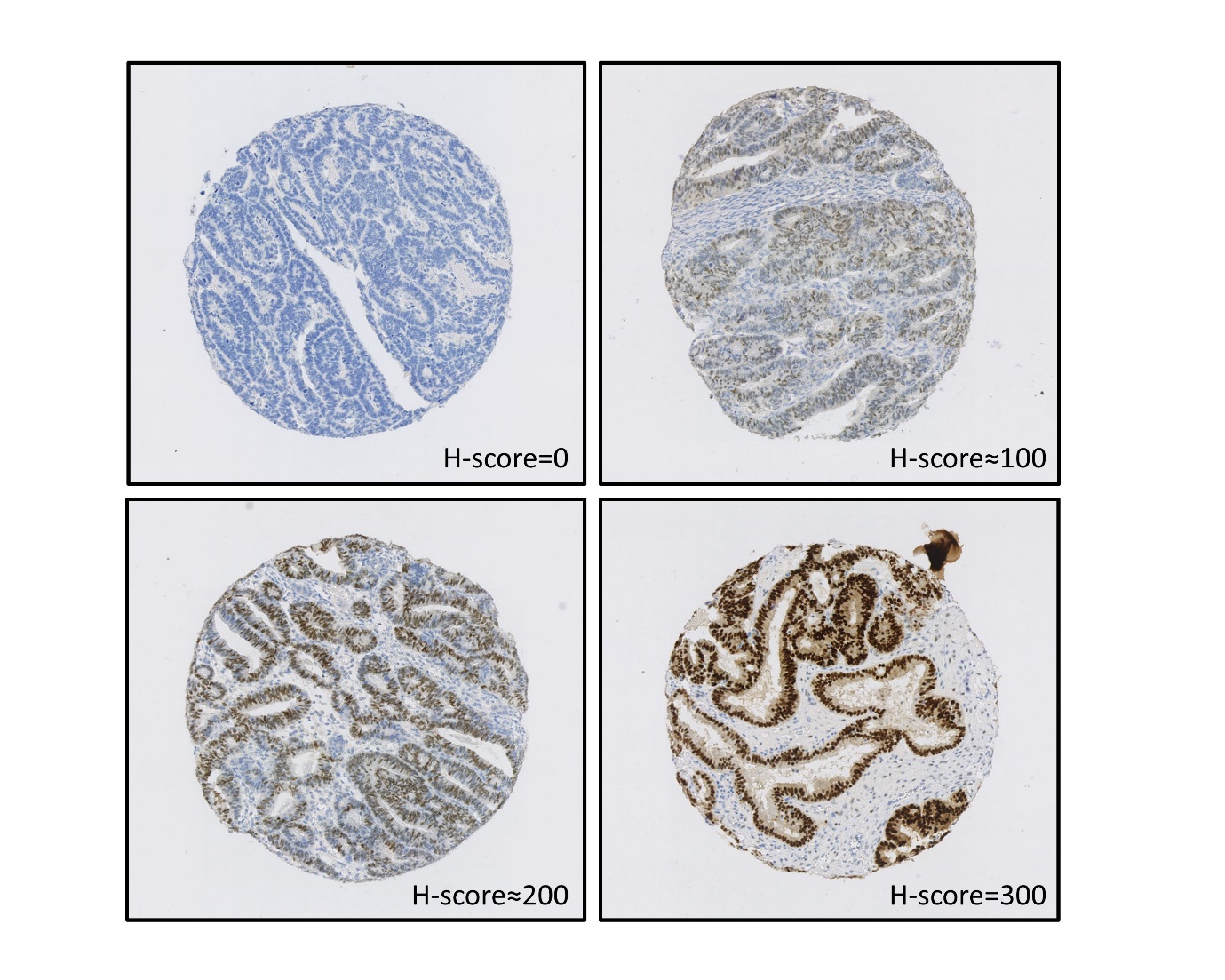


Figure S1. Histoscore examples.


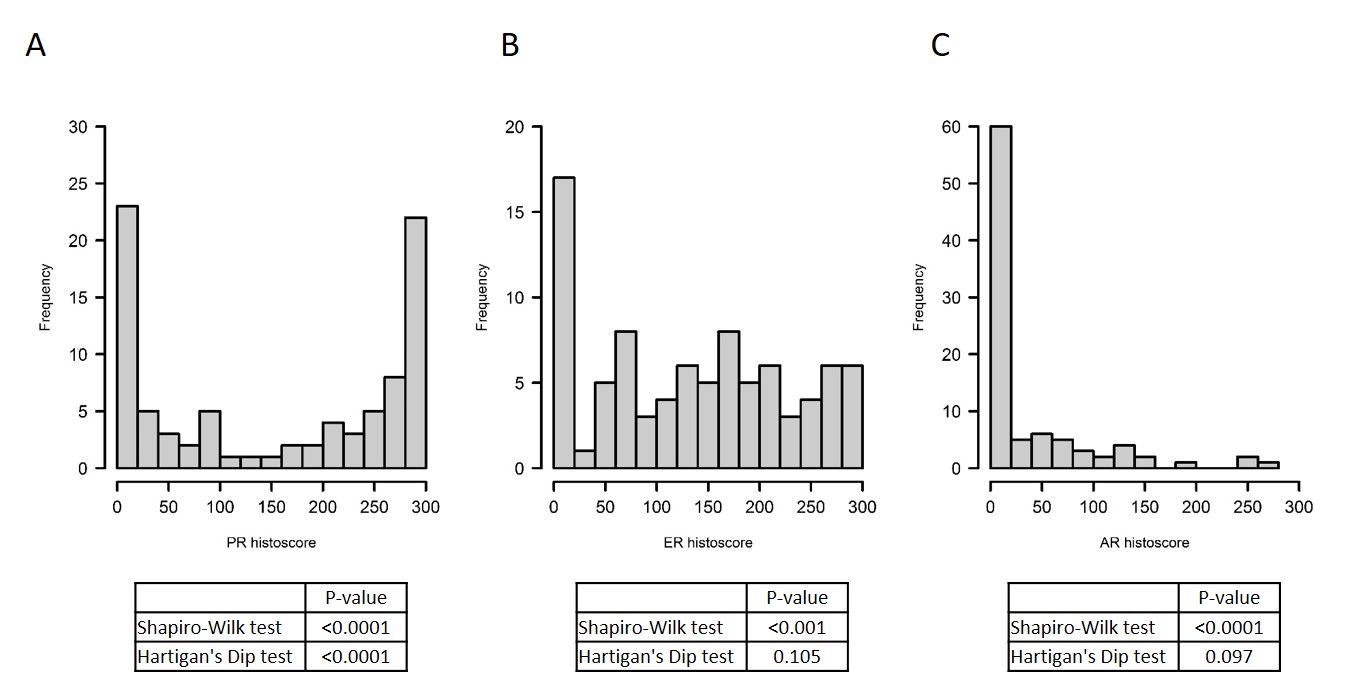


Figure S2. Distribution of PR, ER and AR expression across EnOCs.


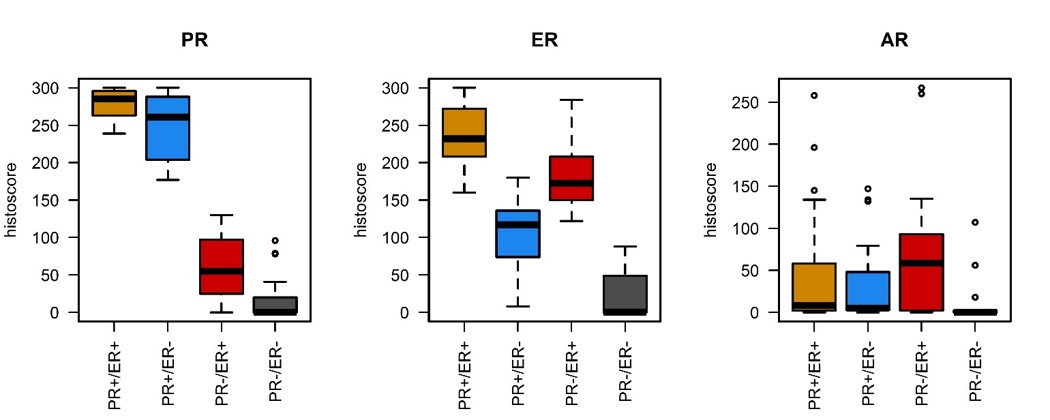


Figure S3. PR, ER and AR histoscores across EnOC subgroups identified by hierarchical clustering


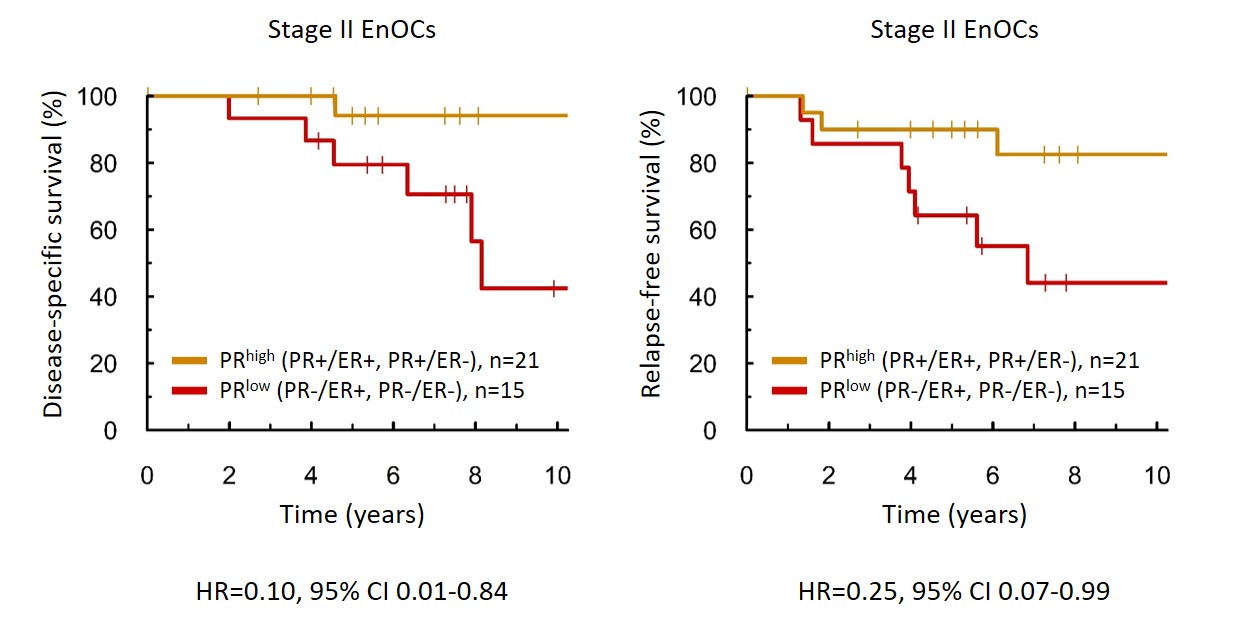


Figure S4. Clinical outcome of stage II EnOC patients in PR^high^ (PR+/ER+ and PR+/ER-) and PR^low^ (PR-/ER+ and PR-/ER-) groups.

Table S1. Adjuvant therapy and staging for EnOC cases.

|  | | Stage I^a^  (N=47) | | Stage II^b^ (N=42) | | Stage III/IV (N=16) | | Unknown Stage (N=2) | |
| --- | --- | --- | --- | --- | --- | --- | --- | --- | --- |
|  |  | n | % | n | % | n | % | n | % |
| Adjuvant therapy | Surgery only | 24 | 51.1 | 3 | 7.1 | 3 | 18.8 | 0 | 0.0 |
|  | Single-agent platinum | 13 | 27.7 | 15 | 35.7 | 8 | 50.0 | 2 | 100.0 |
|  | Platinum-taxane combination | 2 | 4.3 | 17 | 40.5 | 1 | 6.3 | 0 | 0.0 |
|  | Other platinum combination | 2 | 4.3 | 3 | 7.1 | 3 | 18.8 | 0 | 0.0 |
|  | Other chemotherapy | 4 | 8.5 | 3 | 7.1 | 0 | 0.0 | 0 | 0.0 |
|  | Radiotherapy | 2 | 4.3 | 1 | 2.4 | 0 | 0.0 | 0 | 0.0 |
|  | Endocrine therapy | 0 | 0.0 | 0 | 0.0 | 1 | 6.3 | 0 | 0.0 |
| Staging | Fully staged^d^ | 32 | 68.1 | 36 | 85.7 | 11 | 68.8 | 0 | 0.0 |
|  | Under-staged^e^ | 12 | 25.5 | 4 | 9.5 | 5 | 31.3 | 2 | 100.0 |
|  | Unknown | 3 | 6.4 | 2 | 4.8 | 0 | 0.0 | 0 | 0.0 |

^a^stage IA, n=18; stage IB, n=1; stage IC, n=28

^b^stage IIA, n=3; stage IIB, n=10; stage IIC, n=27; unknown sub-staging, n=2

^c^inoperable / no surgery, n=4; no omentectomy, n=1

^d^Full surgical staging was defined in our study as total abdominal hysterectomy (TAH), bilateral salpingo-oopherectomy, omental biopsy or omentectomy, and washings. Only 2 patients underwent lymph node sampling, no patients underwent lymphadenectomy. Only 1 patient with stage IA disease underwent adjuvant chemotherapy due to the absence of lymph node sampling.

^e^Patients who were understaged with stage I and II disease included: no omentectomy (n=9), no TAH and omentectomy (n=2), subtotal hysterectomy (n=3), no salpingectomy (n=1), only right oophorectomy performed (n=1).

Table S2. Comparison of histoscoring between observer 1 and observer 2 for PR, ER and AR

| Marker | Weighted kappa statistic | Spearman's concordance correlation |
| --- | --- | --- |
| PR | κ=0.98 | ρ=0.96, P<0.0001 |
| ER | κ=0.96 | ρ=0.97, P<0.0001 |
| AR | κ=0.94 | ρ=0.85, P<0.0001 |

Table S3. Summary of PR, ER and AR histoscores across 107 WT1 negative EnOCs.

|  | N evaluable patients (%) | Mean | Median | Range | Shapiro-Wilk normality test | Hartgian’s Dip test for modality |
| --- | --- | --- | --- | --- | --- | --- |
| PR | 87 (81.3%) | 155.1 | 186 | 0-300 | P<0.0001 | P<0.0001 |
| ER | 87 (81.3%) | 136.5 | 136 | 0-300 | P<0.001 | P=0.105 |
| AR | 91 (85.0%) | 36.0 | 3 | 0-267 | P<0.0001 | P=0.097 |

Table S4. PR, ER and AR histoscores of EnOC subgroups

|  | | Group | | | |
| --- | --- | --- | --- | --- | --- |
|  |  | PR+/ER+ | PR+/ER- | PR-/ER+ | PR-/ER- |
| PR histoscore | median | 285.0 | 261.0 | 55.0 | 0.0 |
|  | range | 239-300 | 177-300 | 0-130 | 0-96 |
| ER histoscore | median | 232.0 | 117.0 | 172.5 | 0.5 |
|  | range | 160-300 | 8-180 | 122-284 | 0-88 |
| AR histoscore | median | 8.0 | 5.0 | 58.5 | 0.0 |
|  | range | 0-258 | 0-147 | 0-267 | 0-107 |

Table S5. Univariable analysis of survival between EnOC subgroups

| Analysis | Subgroup | Univariable HR | 95% CI | P-value |
| --- | --- | --- | --- | --- |
| Disease-specific survival | PR+/ER+ | 0.11 | 0.02-0.54 | 0.006 |
|  | PR+/ER- | 0.05 | 0.01-0.45 | 0.007 |
|  | PR-/ER- | 0.50 | 0.18-1.40 | 0.187 |
|  | PR-/ER+ | reference | reference | reference |
| Relapse-free survival | PR+/ER+ | 0.11 | 0.02-0.51 | 0.005 |
|  | PR+/ER- | 0.24 | 0.07-0.80 | 0.020 |
|  | PR-/ER- | 0.51 | 0.19-1.38 | 0.186 |
|  | PR-/ER+ | reference | reference | reference |

HR, hazard radio; CI, confidence interval

Table S6. Multivariable analysis of disease-specific survival

|  | Factor | HR | 95% CI | P-value |
| --- | --- | --- | --- | --- |
| EnOC subgroup | PR+/ER+ | 0.05 | 0.01-0.35 | 0.002 |
|  | PR+/ER- | 0.05 | 0.00-0.51 | 0.012 |
|  | PR-/ER- | 0.24 | 0.06-0.99 | 0.048 |
|  | PR-/ER+ | reference | reference | reference |
| FIGO stage at diagnosis | I | 0.14 | 0.03-0.66 | 0.013 |
|  | II | 0.37 | 0.09-1.61 | 0.184 |
|  | III/IV | reference | reference | reference |
| Disease grade | G1 EnOC | 0.79 | 0.09-6.79 | 0.834 |
|  | G2 EnOC | 1.42 | 1.18-11.57 | 0.741 |
|  | G3 EnOC | reference | reference | reference |
| Residual disease following debulking | <2cm | 0.04 | 0.00-0.25 | <0.001 |
|  | ≥2cm | reference | reference | reference |
| Age at diagnosis | years | 0.99 | 0.93-1.06 | 0.837 |

HR, hazard radio; CI, confidence interval

Table S7. Multivariable analysis of relapse-free survival

|  | Factor | HR | 95% CI | P-value |
| --- | --- | --- | --- | --- |
| EnOC subgroup | PR+/ER+ | 0.05 | 0.01-0.32 | 0.002 |
|  | PR+/ER- | 0.29 | 0.07-1.20 | 0.088 |
|  | PR-/ER- | 0.25 | 0.07-0.97 | 0.045 |
|  | PR-/ER+ | reference | reference | reference |
| FIGO stage at diagnosis | I | 0.08 | 0.02-0.35 | <0.001 |
|  | II | 0.20 | 0.05-0.84 | 0.029 |
|  | III/IV | reference | reference | reference |
| Disease grade | G1 EnOC | 0.87 | 0.12-6.11 | 0.888 |
|  | G2 EnOC | 2.23 | 0.33-15.10 | 0.410 |
|  | G3 EnOC | reference | reference | reference |
| Residual disease following debulking | <2cm | 0.03 | 0.00-0.21 | <0.001 |
|  | ≥2cm | reference | reference | reference |
| Age at diagnosis | years | 0.99 | 0.94-1.04 | 0.574 |

HR, hazard radio; CI, confidence interval
